# Optimization of Piezoresistive Strain Sensors Based on Gold Nanoparticle Deposits on PDMS Substrates for Highly Sensitive Human Pulse Sensing

**DOI:** 10.3390/nano12132312

**Published:** 2022-07-05

**Authors:** Yu-Shun Su, Wei-Rong Yang, Wei-Wun Jheng, Watson Kuo, Shien-Der Tzeng, Kiyokazu Yasuda, Jenn-Ming Song

**Affiliations:** 1 Department of Materials Science and Engineering, National Chung Hsing University, Taichung 402, Taiwan; zack10512@gmail.com (Y.-S.S.); g110066113@mail.nchu.edu.tw (W.-R.Y.); 2 Department of Physics, National Chung Hsing University, Taichung 402, Taiwan; cyril821129rs@gmail.com (W.-W.J.); wkuo@phys.nchu.edu.tw (W.K.); 3 Department of Physics, National Dong Hwa University, Hualien 974, Taiwan; sdtzeng@gms.ndhu.edu.tw; 4 Division of Materials and Manufacturing Science, Graduate School of Engineering, Osaka University, Osaka 565-0871, Japan; yasuda@mapse.eng.osaka-u.ac.jp; 5 Innovation and Development Center of Sustainable Agriculture, National Chung Hsing University, Taichung 402, Taiwan; 6 Smart Sustainable New Agriculture Research Center, National Chung Hsing University, Taichung 402, Taiwan

**Keywords:** nanoparticle, PDMS, piezo-resistance, gauge factor, arterial pulses

## Abstract

In this study, highly-sensitive piezoresistive strain sensors based on gold nanoparticle thin films deposited on a stretchable PDMS substrate by centrifugation were developed to measure arterial pulse waveform. By controlling carbon chain length of surfactants, pH value and particle density of the colloidal solutions, the gauge factors of nanoparticle thin film sensors can be optimized up to 677 in tensile mode and 338 in compressive mode, and the pressure sensitivity up to 350. Low pH and thin nanoparticle films produce positive influences to superior gauge factors. It has been demonstrated that nanoparticle thin film sensors on PDMS substrates were successfully applied to sense arterial pulses in different body positions, including wrist, elbow crease, neck, and chest.

## 1. Introduction

The principle of commercial pulse diagnosis devices involves attaching a radial sensor to the artery position, where the arterial pulses introduce stress or strain to the sensor, causing periodic voltage change or resistance change [1]. The pulse wave can thus be transduced and recorded in the form of electrical signals. In order to enhance waveform resolution, the improvement of sensor sensitivity is still an ongoing issue.

**Figure 1:**
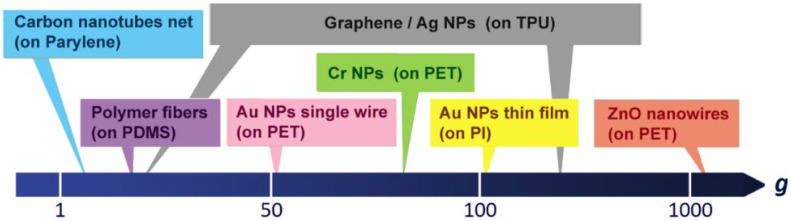
Comparison of gauge factors of several nanomaterials-based strain sensors [2,3,4,5,6,7,8,9].

Among the technologies for tactile sensors, piezo-resistance devices [2], in which the electrical resistance changes due to stress (or strain), are good candidates for such applications. Conventional piezoresistive materials, e.g., metal foils, show very low gauge factor (2~3). Barium titanate and Si-based semiconductors exhibit high gauge factor (>100) but poor flexibility, which may not be suitable for human motion sensing applications. In recent years, various kinds of nanomaterials have been used, developed as strain sensors (Figure 1), such as polymer nanofibers (on polydimethylsiloxane, PDMS) [3], nano-carbon nets (on parylene) [4], nano-chromium films (on polyethylene terephthalate, PET) [5], graphene/Ag nanoparticles (NPs) (on thermoplastic polyurethane, TPU) [6], nano-gold single wires (on PET) [7], Au nanoparticles (NPs) thin film (on polyimide, PI) [8], and ZnO nanowires (on PET) [9]. As also shown in Figure 1, the graphene/AgNP sensors possess a gauge factor (*g*) of 476 at 500% strain but only of 7 at 50% strain. The ZnO-nanowires sensors exhibit a superior *g* value of up to 1813.

Over a decade, intensive efforts have been devoted to building flexible sensors using conductive nanomaterials based on piezoresistive mechanisms. In particular, highly sensitive tactile sensors can be made quite effectively with gold nanoparticles [10,11]. Compared to nanoparticles of other metals [5,12], there are several benefits to choosing AuNPs in the following aspects. AuNPs, as widely known, can be easily synthesized with a well-controlled and uniform size by reduction of HAuCl_4_ [13]. There are possible routes for replacement of the molecule encapsulating AuNPs with a different molecule length and functional group [14]. It is easy to assemble long-range-ordered monolayer or multi-layers of AuNPs by several low-cost methods, such as Langmuir−Blodgett [15], solvent evaporation [16], electrostatic methods [17], entropy-driven assembly [18], and centrifugal deposition [19]. The interparticle distance can be finely tuned by the lengths of ligand or linker molecules, resulting in electrical and optical properties for one’s need [20]. In addition, devices based on surface-modified AuNPs are stable and durable in their flexible and stretchable usage.

Due to the quantum mechanism of electron transport between nanoparticles, films comprising nanoparticles can act as piezoresistive layers and thus strain gauges [11,21,22,23,24,25]. The interparticle distance changes caused by applied strain result in tunneling probability change, consequently affecting the conductance of electrons. The large resistance change arises from the strain-sensitive hopping conduction in these NP films, based on the conductance formula in the weak coupling regime at high temperatures [21,26]

(1) $$G=G_{0}e^{-\beta s}e^{-E_{a}/k_{B}T}$$

where *s* is the interparticle spacing, and *β* is a constant, typically 9~13 nm^−1^, related to free space tunneling or molecule chain hopping as the interparticle conduction mechanism [27]. *E_a_* stands for thermal activation energy for the single particle Coulomb charging effect. At room temperature, *E_a_* is usually smaller than *k*_B_*T* (~26 meV when *T* is around 300 K), thereby the factor *e*^−*βs*^ is dominating. Equation (1) only gives a microscopic and qualitative description for the conductivity based on molecule junctions. For a real sensor, there are other collective effects, such as crack junction [28] and percolations [29,30].

Taking advantage of the flexibility and stretchability of PDMS substrates, Au NP thin film sensors on PDMS substrates exhibiting *g* values higher than 100 have been successfully prepared by centrifuging method, and demonstrated to be capable of monitoring human pulses [31]. Compared with other tactile sensors, Au NP-thin films on PDMS show the advantages of high responsibility for pressure with a frequency bandwidth of kHz and nearly isotropic piezoresistive responses. The previous report [31] provided the general scheme to build flexible sensors using Au NPs, and explored their practical usages. To optimize the sensor fabrication for a better sensitivity regarding to the strain and pressure sensing, this study investigates the material parameters for Au NP preparation and film deposition, including surfactants, particle concentrations and pH values of the colloidal solutions. To evaluate the feasibility for practical applications, the sensors thus obtained will be subjected to bending tests, pressure tests, and human pulse measurements at different positions to characterize their functions.

## 2. Experimental Procedures

### 2.1. Preparation of AuNP Thin Films on PDMS

Monodispersed gold nanoparticles were prepared by the reduction of aqueous hydrogen tetra-chloro-aurate (HAuCl_4_, Sigma Aldrich, Burlington, MA, USA) with potassium carbonate (K_2_CO_3_, Sigma Aldrich), trisodium citrate (C_6_H_5_Na_3_O_7_, Sigma Aldrich) and tannic acid (C_76_H_52_O_46_, Sigma Aldrich, Burlington, MA, USA) using a standard procedure [13]. Two surfactants were respectively adopted in the synthesis, i.e., 11-mercaptoundecanoic acid (MUA, HS(CH_2_)_10_CO_2_H) and 3-mercaptopropionic acid (MPA, HS(CH_2_)_2_CO_2_H), both of which have a negative ionic end group. To form Au NP films, acidic Au NP colloidal solution (12 mL) was placed in a 50 mL centrifuge tube of which the inner surface was attached to PDMS substrate using acrylic resin. The pH value and Au NP concentration of the colloidal solutions were controlled. After being centrifuged at 10,000× *g* rpm for 20 min, Au NPs were condensed and deposited onto PDMS (polydimethylsiloxane) substrate via centrifugal force. Before attaching to the centrifuge tube, PDMS substrates were modified by APTMS (3-aminopropyltrimethoxysilane) for which the end of molecules has a mono-positive charge, allowing strong electrostatic attraction to be developed between Au NPs and PDMS.

### 2.2. Assembly of Strain Sensors

**Figure 2:**
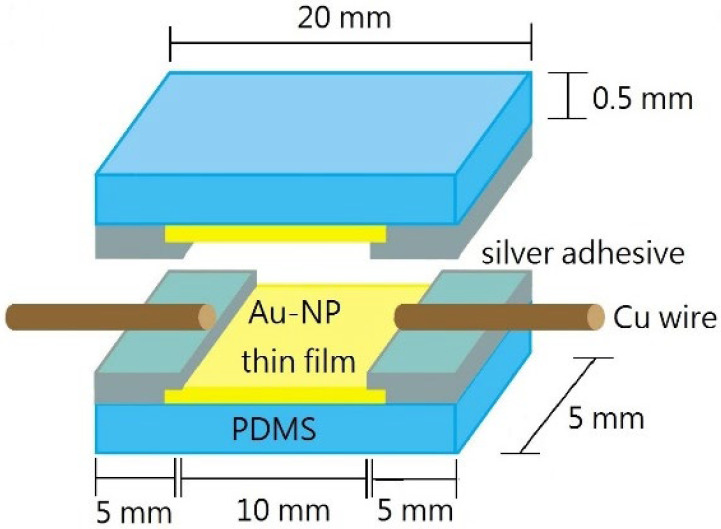
Schematic diagram of the NP thin film strain sensor structure.

The PDMS substrates with Au NP thin films were cut into pieces with a size of 20 mm × 5 mm. As shown in Figure 2, commercial silver adhesives were coated on the two ends of Au NP thin film as electrodes, and two copper wires were stuck into the adhesives. The PDMS was further covered and joined together with another Au NP thin film-coated PDMS substrate.

### 2.3. Characterization Measurement

**Figure 3:**
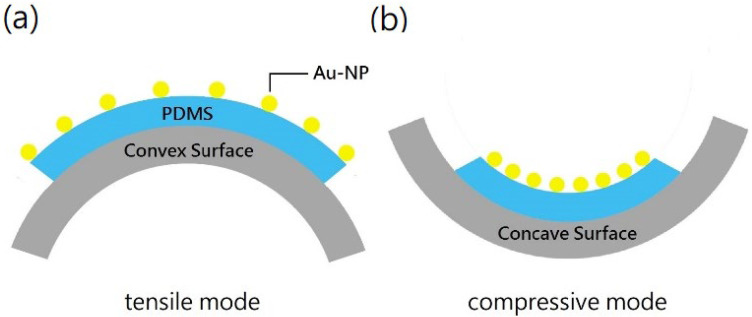
Sketch of two different modes for bending test: (**a**) tensile, and (**b**) compressive.

The microstructures of Au NPs and the thin films were characterized with TEM (JEM-1400, JOEL Ltd., Tokyo, Japan) and FE-SEM (Zeiss UltraPlus, Carl Zeiss Co., Ltd., Oberkochen, Germany), respectively. The UV-visible spectra of the NP solutions were measured by a UV-Vis spectrometer (Jasco V-670, Easton, MD, USA) with a 10 mm quartz cell. The resistance and piezoresistive sensitivity were inferred from the current-voltage measurement using a bias voltage of 0.1 V. The sensors were connected to a homemade electronic amplifier and multimeters, and the electronic signals were recorded using LabVIEW program. The sensors were bended on the surfaces of cylinders with different radii of curvature. As illustrated in Figure 3, the thin film experienced tensile strain when being bended on a convex surface (Figure 3a), and compressive strain on a concave surface (Figure 3b). Applied strain ranged from −0.78% to 0.72%, which was approximately calculated by *ε* = *t*/2*d*, where *t* is the thickness of substrate (0.5 mm) and *d* is radius of curvature.

**Figure 4:**
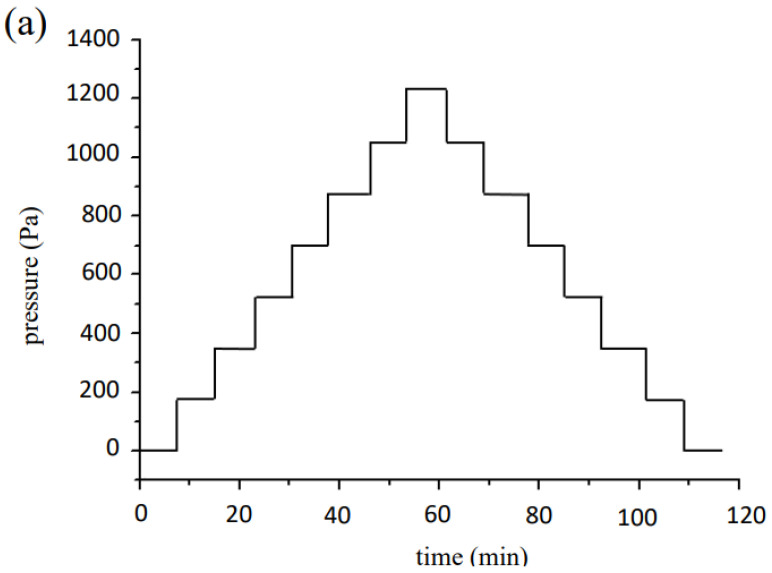

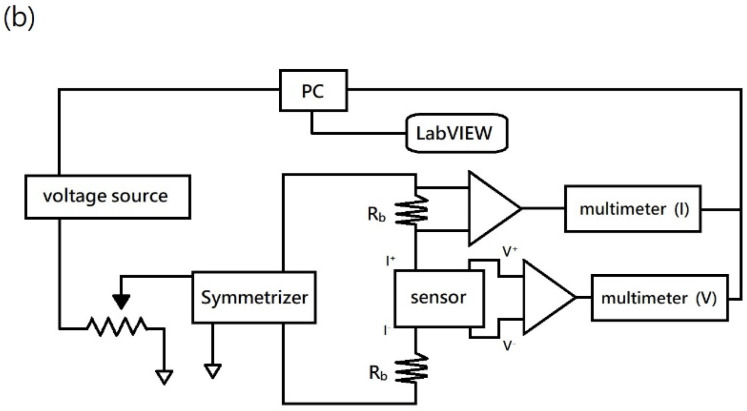
(**a**) Variation of applied pressure during the pressure test; (**b**) pulse measurement setup.

As displayed in Figure 4a, pressure sensitivity was evaluated by subjecting the sensors to normal stresses. The applied load increased step by step by intermittently stacking polymer pieces (5 mm^2^ in cross sectional area and 1.8 g in weight) onto a sensor, and unloading in the same manner afterward. Figure 4b shows the setup of human pulse measurement. During pulse measuring, the strain sensor was attached to the skin above the artery and fastened with a wristband. When a constant voltage bias inputted into the sensor, the output current would change with time and be recorded by computer in real time.

## 3. Results and Discussion

### 3.1. Resistance of MUA Devices and MPA Devices

**Figure 5:**
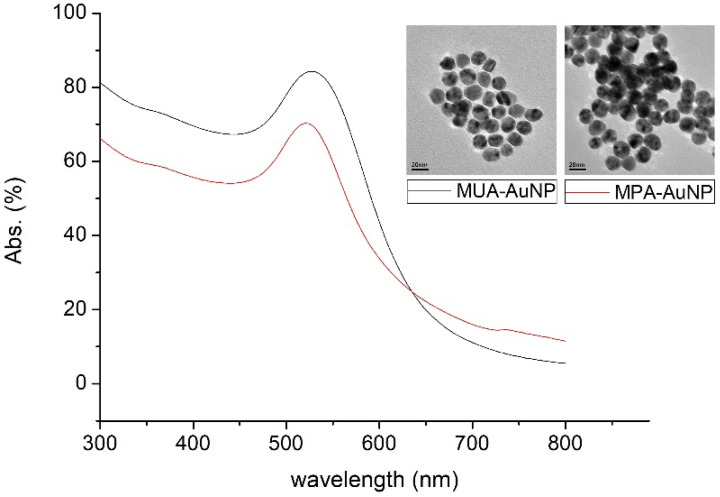
UV-Vis spectra and TEM images of MUA-AuNPs and MPA-AuNPs (red line: MPA, black line: MUA).

Figure 5 shows the UV-Vis spectra and TEM images of Au nanoparticles. The diameter of MUA-protected Au NPs was estimated to be 17.5 ± 3.7 nm, while that of MPA-protected Au NPs was 16.2 ± 4.0 nm. A distinct absorption peak could be seen at a wavelength of around 520 nm.

**Figure 6:**
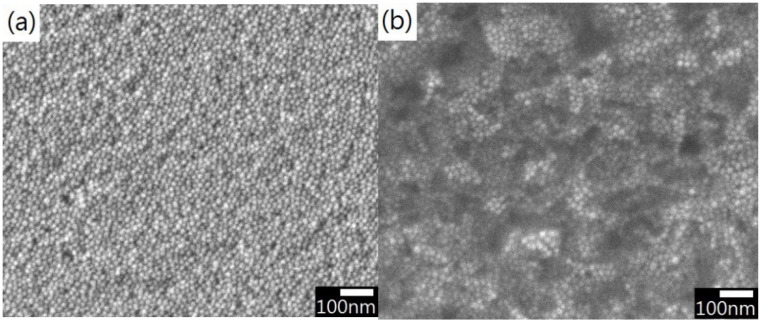
MUA-Au nanoparticle films: (**a**) pH = 3.5 (**b**) pH = 3.2.

Taking MUA Au NPs, for instance, centrifugally-deposited Au NP films show a uniform manner using colloidal solutions with pH value of 3.5 and NP concentration of 1.32 × 10^13^ mL^−1^ (Figure 6a), but the surface was relatively rugged when pH was slightly decreased to 3.2 (Figure 6b). This might affect the electrical performance of the deposited films.

**Figure 7:**
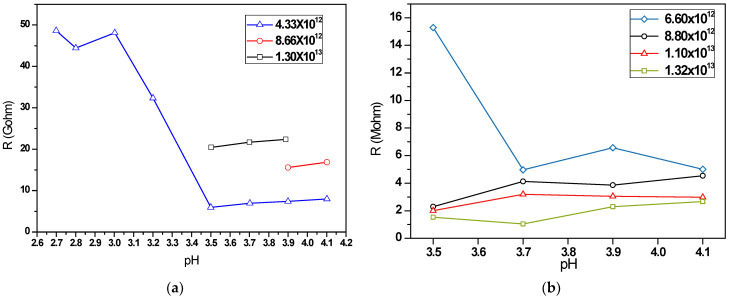
Electrical resistance of NP devices: (**a**) MUA devices; (**b**) and MPA devices.

As shown in Figure 7a, the assembled MUA devices possessed electrical resistances ranging from 6 to 50. Using colloidal solution with NP concentration of 1.32 × 10^13^ mL^−1^, when pH was less than 3.5, the film resistances were about five times greater than those of 3.5 and above. This can be ascribed to the stronger agglomeration tendency of Au nanoparticles in high acidic environment, which gave rise to a larger amount of nanoparticle clusters formed in the solution. Those discrete lumps then stuck to the PDMS and caused an uneven structure, as well as inferior electrical conductance. With a higher particle concentration, the resistance of the coated films was getting higher or even undetectable at low pH values. Because of the extremely high impedance which might lead to a low signal-to-noise ratio in practice, MUA-Au NPs are not good choices for sensor applications.

As for MPA-Au NPs, Figure 7b illustrates the variation in electrical resistance of thin films fabricated using colloidal solutions with pH of 3.5~4.1 and particle concentrations of 6.6 × 10^12^~1.32 × 10^13^ mL^−1^, corresponding to film thickness of 119~238 nm. Compared to MUA NP devices, the assembled MPA NP devices possessed a much lower resistance ranging from 1 to 16 MΩ. The difference could be attributed to the length of surfactant molecules. MUA was based on a 11-carbon chain, and MPA on a 3-carbon chain [32]. Although an acid of a longer alkyl chain is more capable of preventing nanoparticle aggregation and enhancing stability [33], it is found that MUA (11-mercaptoundecanoic acid) is too long to provide a usable device. On the other hand, the protective layer on MPA-AuNPs was much thinner and thus a shorter tunneling barrier for electrons. Figure 7b also reveals that the resistance of sensors decreased with higher particle concentration (or greater film thickness), and the effect of pH value was negligible except for those with NP concentration of 6.6 × 10^12^~1.32 × 10^13^ mL^−1^. Considering the proper resistance range, MPA-AuNP devices were adopted for sensing of pressure, strain and pulses.

### 3.2. Gauge Factor of MPA Devices

**Figure 8:**
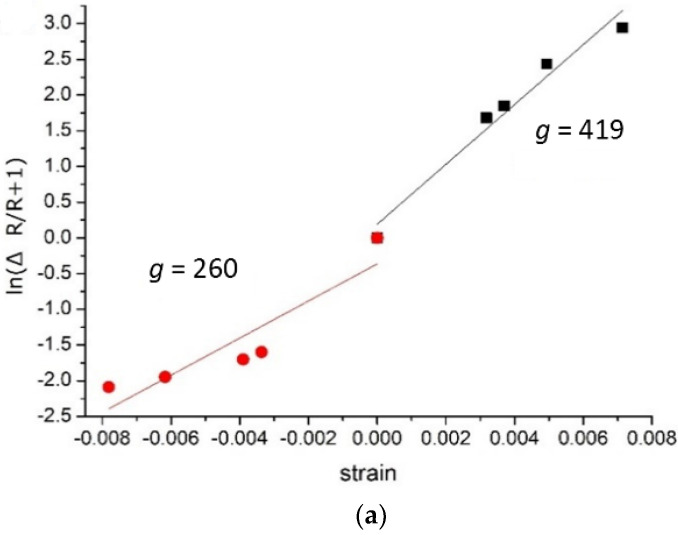

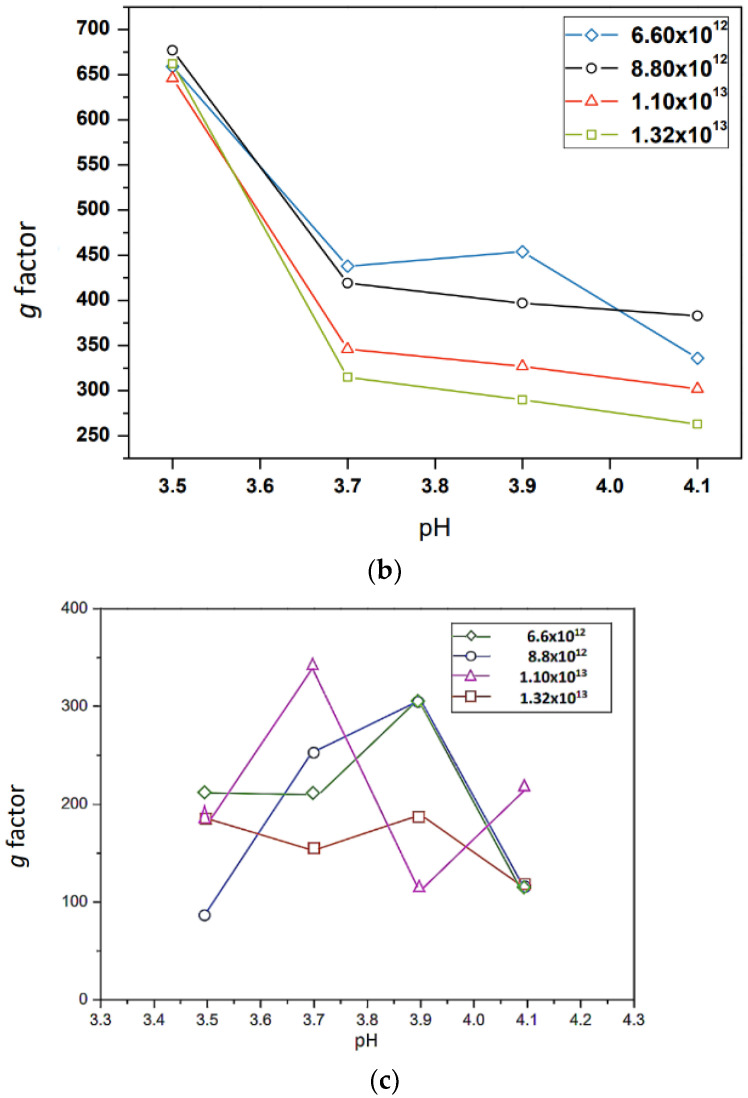
(**a**) Relative change of resistance with strains and the *g* factors, (**b**) tensile-mode and (**c**) compressive-mode gauge factors of MPA devices.

In order to evaluate the piezoresistive sensitivity of the NP thin film devices, MPA sensors were bent with various strains. The gauge factor could be obtained by the following equation:

*g* = *ln*(Δ*R*/*R* + 1)/*ε* (2)

where Δ*R*/*R* is the ratio of electrical resistance change and *ε* is the strain. Subjected to strain from −0.78% to 0.72%, the change of electrical resistance reached 100~3000% under tensile strain, but was lower than 100% under compressive strain. When a MPA sensor fabricated using colloidal solutions with pH of 3.7 and particle concentration of 8.8 × 10^12^ mL^−1^ was subjected to strain from −0.78% to 0.72%, the tensile-mode gauge factor was 419, and the compressive-mode gauge factor reached 260 as illustrated in Figure 8a. The tensile-mode gauge factors for all the pH and NP concentration conditions are summarized in Figure 8b, indicating the *g* factors ranged from 263 to 677. High NP concentration as well as high pH led to smaller *g* factors under tension. Figure 8c illustrates that no distinct relationship between *g* factor and pH value could be found. The compressive-mode gauge factor was smaller than those in the tensile mode and ranged from 90 to 338. It can be inferred that in the compression mode there may be other effects affecting the variation of gauge factor.

### 3.3. Pressure Sensitivity of MPA Devices

**Figure 9:**
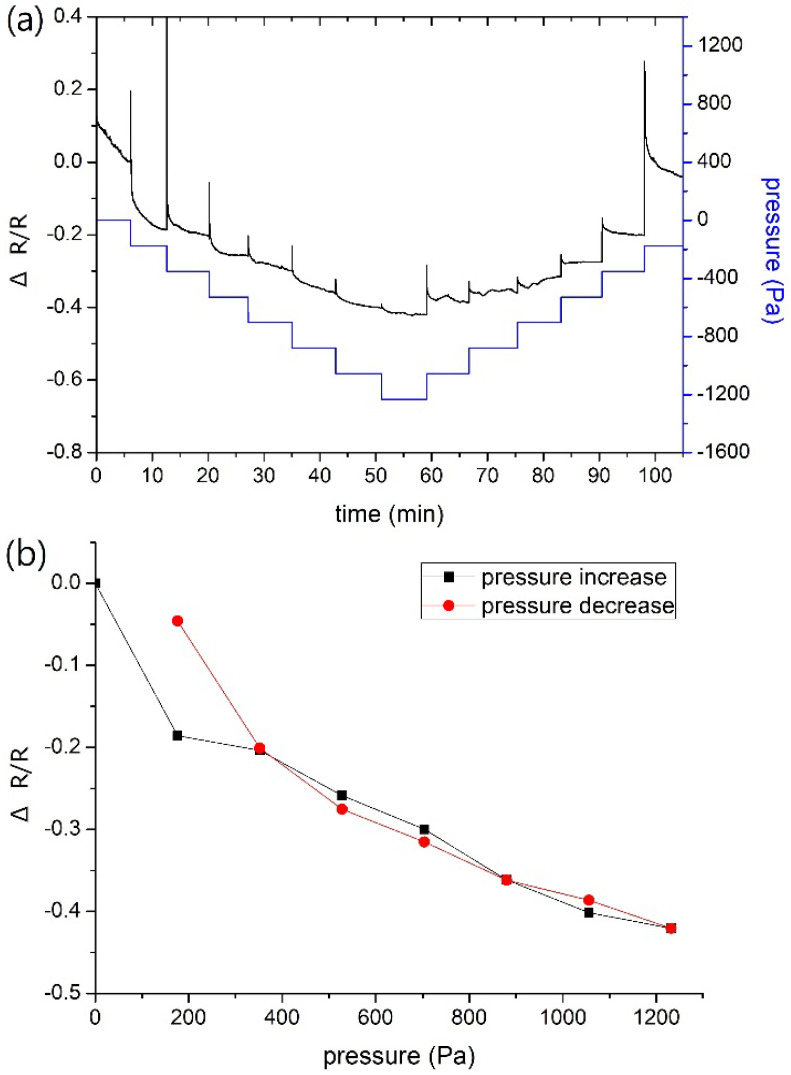
(**a**) Change of resistance with time during the pressure test; (**b**) change of steady values of resistance with applied pressure.

Figure 9a shows the resistance change of the MPA sensor fabricated with the conditions of NP concentration 6.6 × 10^12^ mL^−1^ subjected to increasing and then decreasing pressure loadings, and the relationship between electrical resistance and the pressure is illustrated in Figure 9b. The applied pressure varied step-by-step at intervals of 176 Pa as indicated by the blue line (Figure 9a). The resistance overshot as pressure changed suddenly, and recovered to a steady state. The steady values of resistance descended with increasing pressure (Figure 9b), and vice versa. The average pressure sensitivity was evaluated using the following equation [33]:

*S* = (Δ*R*/*R*)/Δ*P* (3)

where Δ*P* and Δ*R* are the changes of pressure and resistance, respectively. Accordingly, *S* of 350 MPa^−1^ can be calculated. Since *S* of 160 MPa^−1^ could be obtained when the sensor was fabricated with NP concentration of 8.8 × 10^12^ mL^−1^, it can be surmised that pressure sensitivity also depends on the particle concentration.

### 3.4. Pulse Wave Measurement with MPA Devices

**Figure 10:**
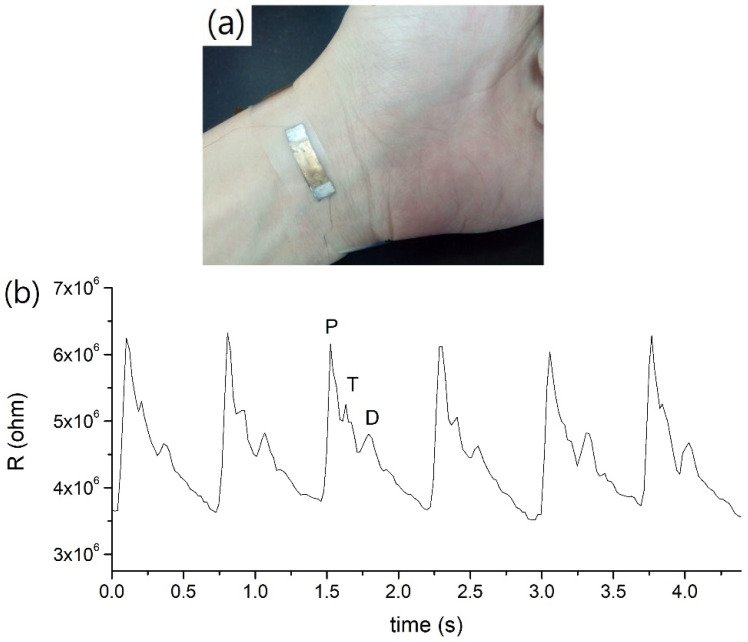
(**a**) The picture of an MPA sensor monitoring pulse at wrist; (**b**) the pulse waveform.

**Figure 11:**
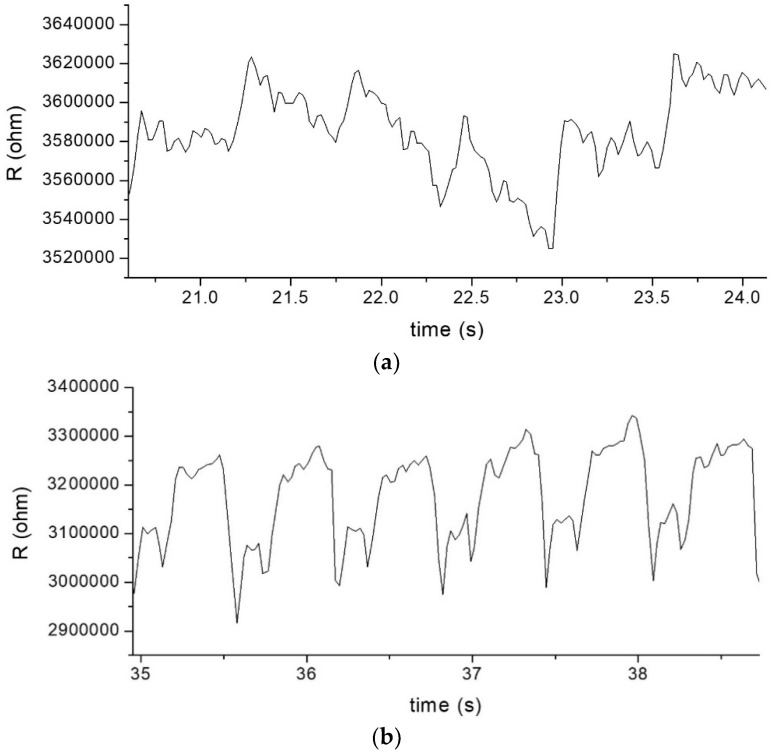

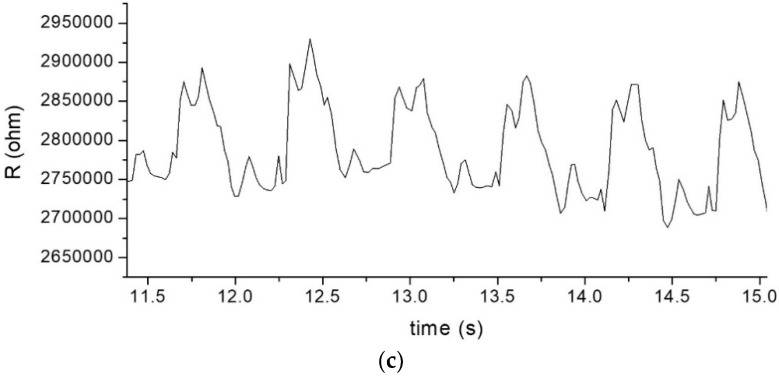
Demonstration of strain sensors palpating pulses at different body parts: (**a**) elbow crease, (**b**) neck, and (**c**) chest.

Figure 10 and Figure 11 show the results of pulse measurement on the same person by using MPA sensors with tensile-mode *g* factor of 419 and compressive-mode *g* factor of 260. When the sensor was fastened on the wrist skin (Figure 10a), the arterial pulse would cause periodic changes of resistance, which were recorded with a frequency of about 50 points per second. As illustrated in Figure 10b, three major crests could be recognized in one pulse wave. In order of peak intensity, they are P wave (or main peak), T wave (or pre-dicrotic peak), and D wave (dicrotic peak) [1], respectively. Figure 11 shows the pulse waves measured from other body positions, including elbow crease (Figure 11a), neck (Figure 11b), and chest (Figure 11c), by using the same sensor. Not all three peaks can be easily identified. The T waves were not always distinct. The P waves and D waves could be recognizable in most of the cases, while the T waves were often merged into P waves.

Moreover, the relative changes in resistance shown in Figure 11a–c are 1.0%, 8.0% and 3.7%, respectively. They are apparently much smaller than the value of 50% when monitoring at the wrist position (Figure 10b). Nevertheless, the resistance change at this level can still be easily measured and the signal can be processed to provide the heart rate (such as by fast Fourier transform). The characteristics of the signals recorded from the chest (Figure 11c) are similar to those on th3 electrocardiogram (ECG) waveform [34]. The explanation of the pulse characteristics related to the physical conditions needs further investigation.

## 4. Conclusions

Strain sensors based on Au NP thin films on stretchable PDMS substrates were successfully fabricated using centrifugal method, and the Au NPs were protected by MUA and MPA molecules respectively. The MUA-Au NP sensors had extremely high electrical resistance of 10^9^~10^10^ Ω and cannot be used in practice. The MPA Au NP sensors possessed resistance of 10^6^~10^7^ Ω tensile-mode gauge factors ranging from 263 to 677, compressive-mode gauge factors ranging from 90 to 338, and pressure sensitivity ranging from 160 to 350. Experimental results suggest that low pH and thin NP films brought about superior gauge factors. The MPA sensors exhibited outstanding piezoresistive sensitivity compared to other currently existing strain sensors. In the pulse wave measurement at the wrist, the waveforms consisting of three major crests can clearly be detected by the MPA sensors. It has been demonstrated that an MPA sensor could also be applied to sense pulses in other body positions, including elbow crease, neck, and chest.

## Data Availability

Not applicable.
